# Association between dietary patterns and pregnancy induced hypertension: a case control study from Sudan

**DOI:** 10.4314/ahs.v22i4.42

**Published:** 2022-12

**Authors:** Kambal Nahla, Bani A Ibrahim, E A Rahim Bahaa-eldin

**Affiliations:** 1 Jazan University, Faculty of Applied Medical Sciences, Department of Clinical Nutrition; 2 Ajman University, College of Medicine; Ajman University, Center of Medical and Bio-allied Health Sciences Research; 3 Independent Researcher, OKC, The United States

**Keywords:** Nutrition, pregnancy-induced hypertension, health, Sudan

## Abstract

**Background:**

Dietary patterns and its associated factors and can play an essential role in development of preeclampsia and implication in pregnancy-induced hypertension (PIH).

**Objective:**

This study was performed to determine nutritional factors associated with PIH among pregnant women in Khartoum, Sudan.

**Methods:**

This study was a case-control involving 100 women with PIH and 200 normotensive pregnant women.

**Results:**

The mean current weight in the control and PIH groups was 70.25 ± 12.35 and 72.42 ± 12.33 kg; whereas the mean energy intake in the control and PIH groups was 1991.58 ±140.72 and 2154.37 ± 130.85 kcal, respectively. The study results indicated a significantly lower risk of PIH among women ingesting fruits and legumes (odds ratio, 8.44 and 4.07, respectively; 95% confidence interval; P < 0.05).

**Conclusion:**

PIH was positively associated with higher energy, fat, and protein intake. A lower risk of PIH was calculated for women whose dietary habits included fruits and legumes. Measures with which to ensure proper nutrition education are needed to obtain good health and pregnancy outcomes. PIH is a multifactorial disease with an unclear etiology, and the findings of this study will serve as a baseline for future studies in this field.

## Introduction

Pregnancy-induced hypertension (PIH) is a common and dangerous disorder in pregnancy that occurs in the absence of other causes of elevated blood pressure, and it increases both the child's and mother's risk of morbidity and mortality 1–2. PIH accompanied by gestational hypertension, together with preeclampsia and eclampsia, is described as an abnormal rise in blood pressure that usually develops after the 20^th^ week of pregnancy. Other than hypertension, symptoms of preeclampsia include proteinuria and edema. If this critical situation progresses to eclampsia, life-threatening convulsions and coma may occur. The consequences of PIH include low-birth-weight infants and preterm labor and delivery^3^. A systematic review and meta-analysis focusing on the relationship of dietary factors with gestational hypertension and pre-eclampsia showed a higher risk of certain complications associated with pre-eclampsia among pregnant women who had a dietary pattern characterized by processed meat, salty snacks, and sweet drinks 4. PIH is an umbrella that covers a set of disorders including pre-existing and gestational hypertension, preeclampsia, and eclampsia. PIH is responsible for causing complications in approximately 6% to 10% of pregnancies and associated treatment plans; therefore, it is considered a major risk factor for maternal and perinatal morbidity and mortality 1,5–7. An overview on the pathophysiologic developments accompanying pre-eclampsia indicated that the etiology of pre-eclampsia does not involve one single mechanism but is instead considered a cascade of succeeding events; thus, this condition is clearly not ideal for pregnancy 8. In a prospective study involving 3187 Dutch pregnant women, Timmermans et al.^9^ assessed the relationships of dietary patterns with systolic blood pressure (SBP) and diastolic blood pressure (DBP) during pregnancy. They found that low and high compliance with a Mediterranean diet and traditional dietary pattern, respectively, was associated with higher blood pressure during pregnancy. Both dietary factors and the nutritional status, which have been suggested to play a role in the development of preeclampsia, are considered potential risk factors 10. These hypotheses have been insufficiently tested in trials according to an earlier statement 11. Singh et al.^12^ studied the potentiality of a dietary effect on 225 antenatal women with PIH undergoing antenatal care at the obstetrics and gynecology department of a teaching hospital in India. They found that consumption of visible fat was significantly associated with the different types of PIH. In total, 25% and 16% of the antenatal women consumed visible fat and junk food on a regular basis. In a case-control study (449 women with preeclampsia, 449 controls) conducted by Cao et al.1 in Zhengzhou, China, the uncertainty associated with the capability of the Dietary Approaches to Stop Hypertension (DASH) diet to decrease the risk of preeclampsia was examined using a food-frequency questionnaire. The results revealed a significant association between DASH and preeclampsia, and the data suggested an inverse relationship between commitment to a DASH diet and the odds ratio of preeclampsia.

PIH was investigated among women in Ethiopia 13, and the results suggested that nutritional factors affect the risk of hypertensive disorders of pregnancy. The physiological changes during pregnancy can be detrimentally affected by a poor diet. There is some evidence of the role of nutrient deficiencies in the development of PIH. Although the etiology of gestational hypertension and preeclampsia is still uncertain, evidence suggests that diet might play a role. A case-control study (30 pregnant women, 70 control normotensive pregnant women) was conducted in Kumasi, Ghana to identify the association between geophagia and hypertensive disorders of pregnancy and to measure women's dietary intake and health conditions. The study concluded that the women had noticeably low intake of energy and micronutrients that are essential in the third trimester as the fetal requirements for nutrients increase 14. Elevated total energy and lower magnesium and calcium intake measured during pregnancy were recognized as predictive factors for hypertensive disorders of pregnancy 4.

Mohieldein et al.^15^ stated that PIH is a common condition in Sudanese pregnant women as observed by practicing doctors. According to a bunch of Sudanese reserchers^16^,^17^, preeclampsia and eclampsia are considered the main factors associated with obstetric problems and maternal mortality across Sudan, although there are no published data regarding the prevalence of these disorders in Sudan. Therefore, this study was performed to investigate the relationship between dietary habits and PIH, including eclampsia.

## Materials and Methods

This cross-sectional study was performed in Khartoum State, Sudan in 2016. All pregnant women with PIH who fulfilled the inclusion criteria were included in the study. A consecutive sampling technique was applied to select patients at the study facilities. The sample size was determined using OpenEpi version 3 software (Andrew G. Dean and Kevin M. Sullivan, Atlanta, GA, USA). The case:control ratio was 1:2, the hypothetical proportion of controls with exposure was 40, and the least extreme odds ratio to be detected was 2.5 14,18. Case-control studies are particularly designated for investigation of rare diseases such as PIH. However, recruiting a suitable number of study participants is often difficult, as in the present study. Therefore, increasing the number of controls is a solution that is routinely practiced in such studies 14.

For each PIH case, two controls in the same 5-year age group were selected. This study included 300 pregnant women: 100 pregnant women with PIH (cases) and 200 pregnant women with normotension (controls) admitted to 3 teaching hospitals (Elmogran, Bashair, and Maternal Hospital) (power of 80% and 95% confidence level). Pregnant women aged 15 to 40 years were eligible for the study. Pregnant women with medical conditions such as diabetes, pre-existing hypertension, autoimmune disease, kidney or liver diseases, and systemic diseases were excluded.

Body weight was measured to an accuracy of 0.1 kg using a standard balance scale manufactured by Microlife Corporation (Widnau, Switzerland). The questionnaire included maternal age, parity, and gestational age; the participants' 24-hour dietary recall was analyzed using a food composition table and the nutrient content was calculated accordingly. Dietary habits were evaluated according to dietary intake data retrieved from a quantitative food frequency questionnaire, which inquired about the rate of intake of 27 food items within 4 weeks prior to the day of the interview.

Informed consent for voluntarily participation in this study was obtained from each participant before the interview and after the participant had been informatively briefed about the objectives of the study together with her sustained rights. Data were collected using a structured self-administrated questionnaire. The participants were given assistance in completing the questionnaire if necessary.

Data were quantitatively analyzed using SPSS version 16 (SPSS Inc., Chicago, IL, USA). The analysis included descriptive statistics in terms of mean ± standard deviation. Pearson's chi-square test and analysis of variance were respectively used to compare the differences in significance among frequencies and means. Differences were considered significant at P < 0.05.

## Results

The mean age, number of pregnancies, and number of living children were not significantly different between the control and PIH groups (Table 1). The number of stillbirths was significantly higher in the control than PIH group (1.66 ± 2.42 vs. 0.61 ± 1.48, respectively; P < 0.05). Furthermore, the number of neonatal deaths was significantly higher in the control group (P < 0.05).

**Table 1:** Obstetric History of Healthy 1 (Control) and PIH Groups

Item	Control	PIH	t-test	*P*-value
Age (years)	27.8 ± 52.71	29.69 ± 6.82	1.381	0.172
Number of pregnancies (para)	3.86 ± 2.38	3.29 ± 2.50	1.115	0.269
Number of living children	2.06 ± 1.78	1.50 ± 1.75	1.433	0.156
Number of stillbirths	1.66 ± 2.42	0.61 ± 1.48	2.023	0.049
Number of neonatal deaths	0.76 ± 0.76	0.19 ± 0.40	3.972	0.000
Gestational age (weeks)	18.94 ± 8.46	29.51 ± 8.79	5.815	0.000

Data are presented as mean ± standard deviation.

PIH, pregnancy-induced hypertension.

Weight and blood pressure in the two groups are shown in Table 2. The mean pre-pregnancy weight and current weight were not significantly different between the control and PIH groups. However, the mean SBP and DBP were significantly higher in the PIH group (154.91 ± 18.61 and 100.69 ± 13.22 mmHg, respectively) than in the control group (115.77 ± 7.81 and 37.47 ± 5.73 mmHg, respectively) (P = 0.00).

**Table 2:** Weight and Blood Pressure of Healthy (Control) and PIH Groups

Item	Control	PIH	t-test	*P*-value
Pre-pregnancy weight (kg)	68.38 ± 10.66	72.42 ± 12.33	1.048	0.311
Current weight (kg)	70.25 ± 12.35	74.11 ± 14.16	1.361	0.178
Systolic blood pressure (mmHg)	115.77 ± 7.81	154.91 ± 18.61	11.890	0.000
Diastolic blood pressure (mmHg)	37.47 ± 5.73	100.69 ± 13.22	10.898	0.000

Data are presented as mean ± standard deviation.

PIH, pregnancy-induced hypertension.

### Intake of Macronutrients

The mean energy intake and protein intake were significantly higher in the PIH than control group (P < 0.05) (Table 3). Similarly, higher intake of CHO and fat was observed in the PIH than control group, but the difference was not statistically significant.

**Table 3:** Intake of Macronutrients

Macronutrients	Control	PIH	*P*-value
Energy intake (kcal)	1991.58 ± 140.72	2154.37 ± 130.85	0.000
CHO (g)	239.55 ± 24.69	244.49 ± 24.96	0.344
Protein (g)	57.45 ± 10.42	65.13 ± 16.86	0.006
Fat (g)	72.80 ± 31.78	86.53 ± 39.70	0.062

Data are presented as mean ± standard deviation.

PIH, pregnancy-induced hypertension; CHO, carbohydrates.

### Food Habits

The participants' dietary habits were assessed using the food frequency questionnaire (Table 4). Unlike for vegetables, white meat, and red meat, a significant difference (P ≤ 0.05) was calculated for the consumption of all other food products. Higher intake of wheat-based products, sorghum-based products, rice, and red meat was reported in the PIH group than control group, whereas higher intake of legumes, milk and milk products, fruits, vegetables, and white meat was observed among controls. These results indicated a lower risk of preeclampsia among women whose dietary habits included intake of fruits (odds ratio [OR], 8.44; 95% confidence interval [CI]), vegetables (OR, 4.10; 95% CI), legumes (OR, 4.07; 95% CI), and white meat (OR, 3.09; 95% CI). However, no significant associations were found between the intake of vegetables and white meat (P > 0.05).

**Table 4:** Assessment of dietary habits among the study participants

Item	Group		Frequently		Rarely		Total	Odds ratio	*P*-value
Food items		F	%	F	%	F	%		
Wheat-based products	PIH	98	98	2	2	100	100.0	0.12	0.030
	Control	178	89	22	11	200	100.0		
Sorghum-based products	PIH	85	85	15	15	100	100.0	0.02	0.000
	Control	22	11	178	89	200	100.0		
Rice	PIH	40	40.0	60	60.0	100	100.0	0.14	0.001
	Control	18	9	182	91	200	100.0		
Legumes	PIH	41	41	59	59	100	100.0	4.07	0.002
	Control	148	74	52	26	200	100.0		
Milk and milk products	PIH	85	85	15	15	100	100.0	0.00	0.014
	Control	200	100.0	0	0.0	200	100.0		
Fruits	PIH	66	66	34	34	100	100.0	8.44	0.002
	Control	188	94	12	6	200	100.0		
Vegetables	PIH	89	89	7	11	100	100.0	4.10	0.164
	Control	194	97	6	3	200	100.0		
White meat	PIH	85	85	15	15	100	100.0	3.09	0.056
	Control	196	98	4	2	200	100.0		
Red meat	PIH	100	100	0	0	100	100.0	0.00	--
	Control	200	100	0	0	200	100.0		

PIH, pregnancy-induced hypertension; F, frequency

## Discussion

In the current study, we evaluated maternal dietary intake and investigated the association of various dietary practices with PIH. The PIH group had significantly higher intake of energy and protein (P < 0.05) than the control group. This finding is consistent with that reported by Kazemian et al. 2 who performed a similar case-control study and found a positive relationship between higher energy consumption and gestational hypertension.

According to Schoenaker et al. 4, the higher energy consumption by women with PIH and eclampsia than by healthy women may indicate an imbalance between energy consumption and expenditure, which could in turn lead to obesity, a possible risk factor for hypertensive disorders of pregnancy. Despite the evidence that low protein consumption is related to a greater risk of preeclampsia, the Institute of Obstetricians and Gynaecologists 3 showed that it is secondary to the disease rather than a causal factor.

The current study showed numerous high ORs for PIH associated with dietary intake. The study revealed a significantly lower risk of developing PIH among women who ingest fruits and legumes (OR, 8.44 and 4.07, respectively; 95% CI; P ≤ 0.05). However, women with PIH did not seem to adhere to a specific dietary pattern. Ikem et al.^19^ established a relationship between Western dietary habits (i.e., extensive intake of potatoes, mixed meat, margarine, and white bread) and an elevated risk of PIH. In contrast, a seafood-based diet, which is characterized by particularly high levels of fish and vegetable intake, remarkably decreased the risk of PIH.

Consistent with some previous studies showing that PIH was related to reduced risks of infant mortality and early/later neonatal mortality^20^,^21^, the present study showed significant differences in the mean number of neonatal deaths and the mean gestational age (weeks) between the PIH and control groups (P = 0.000). This finding can be attributed to the fact that PIH is rare in early pregnancy. This study also showed significantly increased SBP and DBP in patients with PIH (P = 0.000).

Preeclampsia can be diagnosed in two ways^22^: (1) SBP of ≥140 mmHg or DBP of ≥90 mmHg on two occasions at a ≥4-hour interval in a previously normotensive patient, or (2) SBP of ≥160 mmHg or DBP of ≥110 mmHg (accordingly, hypertension can be diagnosed within minutes to ensure immediate anti-hypertensive treatment).

## Conclusion

Nutritional intake was observed in the overall study population, and women with PIH had higher energy and protein intake than women with normotension. This study also revealed significantly higher SBP and DBP in women with than without PIH. Although a significantly lower risk of developing PIH among women ingesting fruits and legumes was shown, neither the PIH nor control group seemed to adhere to a specific dietary pattern. Throughout gynecological follow-up, healthcare professionals are responsible for providing pregnant women with information regarding dietary practices that are considered harmful during pregnancy. Additionally, proper nutrition practice should be promoted and highly recommended.
